# A single mutation (V64G) within the RING Domain of Z attenuates Junin virus

**DOI:** 10.1371/journal.pntd.0008555

**Published:** 2020-09-25

**Authors:** Steven J. Hallam, John T. Manning, Junki Maruyama, Alexey Seregin, Cheng Huang, David H. Walker, Juan Carlos de la Torre, Slobodan Paessler

**Affiliations:** 1 Department of Pathology, University of Texas Medical Branch, Galveston, Texas, United States of America; 2 Center for Biodefense and Emerging Infectious Disease, University of Texas Medical Branch, Galveston, Texas, United States of America; 3 Department of Immunology and Microbial Science, Scripps University, La Jolla, California, United States of America; 4 Galveston National Laboratory, Institute for Human Infections and Immunity, Galveston, Texas, United States of America; Oxford University Clinical Research Unit, VIET NAM

## Abstract

Junin virus (JUNV) is a New World arenavirus that is the causative agent of Argentine hemorrhagic fever (AHF). Candid#1 (Can) is a live-attenuated vaccine strain of JUNV that since its introduction has resulted in a marked decrease in AHF incidence within the endemic regions of the Pampas in Argentina. Originally, the viral determinants and mechanisms of Can attenuation were not well understood. Recent work has identified the glycoprotein as the major attenuating factor for Can. The establishment of attenuating strategies based on any of the other viral proteins, however, has not been pursued. Here, we document the role of Can Z resulting in incompatibilities with wild type JUNV that results in decreased growth *in vitro*. In addition, this incompatibility results in attenuation of the virus in the guinea pig model. Further, we identify a single mutation (V64G) in the Z protein that is able to confer this demonstrated attenuation. By establishing and characterizing a novel attenuation strategy for New World mammarenaviruses, we hope to aid future vaccine development for related emerging pathogens including Machupo virus (MACV), Guanarito virus (GTOV), and Sabia virus (SABV).

## Author summary

The continual development of safe, effective vaccines against emerging diseases is one of the greatest challenges facing the scientific community. The New World group of mammarenaviruses contains multiple human pathogens, each capable of causing severe hemorrhagic disease. Among these, only Junin virus has a distributed vaccine. By utilizing this vaccine, we are able to determine vaccine development strategies for related New World viruses that represent an emerging threat. Here we demonstrate that manipulation of the viral Z protein is able to produce an incompatibility that ultimately attenuates the virus. This provides yet another tool for future vaccine development to further global public health.

## Introduction

Junin virus (JUNV) is a member of the genus *Mammarenavirus* within the family *Arenaviridae* in the order *Bunyavirales* [1]. JUNV is further classified as a New World (NW) mammarenavirus within Clade B, which includes also the pathogenic NW mammarenaviruses Machupo (MACV), Guanarito (GTOV), Sabia (SABV) and Chapare virus. These viruses are characterized as select agents and can only be studied in a biosafety level 4 (BSL-4) environment. JUNV is the causative agent of Argentine hemorrhagic fever (AHF), a severe disease in humans resulting in a 15–20% fatality rate, if untreated [2]. AHF is the only arenaviral disease for which a preventive vaccine has been developed. A live-attenuated vaccine, Candid#1 (Can), is available for use in JUNV endemic areas within the Argentinian Pampas. This vaccine was produced by serial passage through guinea pig, mouse brain tissue and cell culture to achieve an attenuated phenotype [3]. Since its introduction in 1990, vaccination with Can has resulted in a marked decrease in incidence of AHF. Clinical studies have demonstrated the vaccine’s safety and efficacy in the vaccinated population [4], but the viral determinants and mechanisms of Can attenuation are not well understood [5,6].

Mammarenaviruses are enveloped viruses with a bisegmented negative strand (NS) RNA genome. Each genome segment, large (L) and small (S), uses an ambisense coding strategy to direct synthesis of two viral proteins whose open reading frames are separated by a non-coding intergenic region (IGR). The L segment encodes the large RNA dependent RNA polymerase (LP) and the small multi-functional Z matrix protein. The S segment encodes the viral nucleoprotein (NP) and the glycoprotein precursor protein (GPC). LP and NP are encoded as early gene products while GPC and Z are late products. As with other viruses with limited proteomic complexity, mammarenavirus proteins can perform multiple functions during the viral life cycle [7].

Replication and transcription of the viral genome is mediated by the virus ribonucleoprotein (vRNP) constituted by the viral genome RNA encapsidated by NP and associated LP. As with other segmented NS RNA viruses, mammarenavirus transcription involves a cap-snatching process [8–10], resulting in viral mRNAs that are capped but not polyadenylated. Following accumulation of NP, the LP is able to read through the intergenic termination sites and produce antigenomic RNA [7]. This in turn leads to the production of GPC and Z and the subsequent transition to the late stages of replication. The Z protein is comprised of three domains: N-terminal domain, Really Interesting New Gene (RING) domain, and a C-terminal domain that encodes canonical late (L) domain motifs. The N-terminal domain has been shown to interact with GPC], while Z interaction with LP and NP involves the participation of the RING domain [12,13]. As with the matrix protein of several others NS RNA viruses, mammarenavirus Z protein has been proposed to lock the vRNP into a non-active form *via* interaction with LP [14]. This halts viral RNA synthesis and promotes assembly of mature infectious particles that bud from the plasma membrane in a process mediated by the L domains of Z [15,16]. Nascent viruses are then able to bud from the host membrane and become infectious [17,18].

The Z protein from the pathogenic Romero (Rom) strain of JUNV differs from Can Z in only two amino acid residues: a valine to alanine change at position 18 (V18A) in the N terminal domain and a valine to glycine change at position 64 (V64G) in the RING domain. Here we demonstrate that Z is an important viral determinant of Can attenuation and that mutation V64G plays a role in attenuating the pathogenic Rom strain through introduction of an artificial incompatibility. Our findings may assist with the development of future live-attenuated vaccine candidates against hemorrhagic fever caused by NW mammarenaviruses.

## Materials and methods

### Cells and viruses

A549, Vero, and Vero E6 cells (all sourced from American Type Culture Collection (ATCC)) were propagated in Dublecco’s modified Eagle’s medium (DMEM) with 10% fetal bovine serum (FBS) and 1% penicillin/streptomycin (P/S). Baby hamster kidney (BHK21) (sourced from ATCC) cells were propagated in modified Eagle’s medium (MEM) with 10% FBS and 1% P/S. Viral stocks used in the studies were produced by infection of Vero E6 cells (MOI of 0.01) and collecting the tissue culture supernatants (TCS) following 4 days. The TCS was then filtered through a 0.45 micron syringe filter and concentrated using an Amicon Ultra-15 centrifugal filter (100,000 NMWL). This centrifugation was performed at 2500 x g for 30 min. All work with virulent viruses and infected animals was performed in the biosafety level 4 (BSL-4) facilities at the University of Texas Medical Branch (UTBM) campus.

### Production of recombinant JUNV (rJUNV)

The production of rJUNV for Romero (rRom), Candid#1 (rCan), rRom/CanZ, and rRom/V64G was performed as previously described [19]. BHK21cells were transfected with plasmids harboring the L and S segments in an antigenomic orientation under the control of a Pol-I promoter, and LP and NP ORFs under the control of a Pol-II promoter. The TCS was collected 4 days post transfection and clarified using a 0.45 micron syringe filter. These stocks were then used to produce the experimental virus samples following the procedure outlined above.

### Growth kinetics and titration

Viral growth kinetics were measured in cultured Vero E6 cells. Cell layers were allowed to reach 80% confluence before being infected by rJUNV (MOI of 0.01) in triplicate. TCS was collected at 24, 48, 72, and 96 hours post infection in addition to a time 0 collection. Viral titers were determined by titration in plaque assay. Vero E6 cells were plated into 12-well culture plates. TCS samples underwent 10-fold dilutions and subsequent addition to the cell monolayers. Plates were incubated for 1 hour at 37°C in an atmosphere of 5% CO_2_. Following incubation, each well was overlaid with a solution of MEM with 2% FBS, 1% P/S, and 0.6% tragacanth gum. Plates were then incubated for 7 days at 37°C and 5% CO_2_. Following incubation, each well was fixed with formaldehyde, and stained with crystal violet. Plaques were then counted and the corresponding titer determined.

### Animal study

Eight-week old female Hartley guinea pigs used in this study were purchased from The Charles River Laboratory. Animal studies were approved (IACUC 1902007) by the Institutional Animal Care and Use Committee (IACUC) at UTMB and performed in accordance with National Institutes of Health BSL-4 guidelines. For telemetry, guinea pigs were anesthetized and subcutaneously implanted with BMDS IPTT-300 transponders from Bio Medic Data Systems, Inc. via a trocar needle.

A total of twenty total guinea pigs were utilized in the study. Four groups of five guinea pigs each were infected with rRom, rCan, rRom/CanZ, or rRom/V64G. Guinea pigs were inoculated with 10^3^ PFU via the intraperitoneal (i.p.) route following anesthesia with an isoflurane precision variable-bypass vaporizer. Recording of death and disease symptoms was done using a standardized scale with the following definitions: development of discoordination, ataxia, encephalitis, seizures (ability to eat and drink), paralysis, hind limb or quadriplegic paralysis (unable to eat and drink). Telemetric body temperature monitoring and body weight measurements were collected for the first 15 days of the study. Animals were euthanized after reaching a humane endpoint of 15% body weight loss or at the end of the study.

### Blood clinical chemistry and hematology

Blood collected from the animals was deposited into tubes containing EDTA. Standard hematologic analysis using a VetScan HM5 analyzer was performed on whole-blood samples following manufacturer’s instructions. Clinical chemical analysis was performed on serum utilizing a VetScan VS2 analyzer following manufacturer’s instructions to obtain a comprehensive diagnostic profile.

### Organ titration and histopathological analysis

Following death or euthanasia, kidney, spleen, liver, lung and brain samples were collected from each animal. Half of each organ was placed in 10% buffered formalin for a minimum of 7 days while the other half was homogenized in 1 mL DMEM with 2% FBS. The homogenized samples were centrifuged at 7000 x g for 15 minutes and the resulting supernatant was collected. The supernatant was then used in plaque assay as described above to determine the viral load for each collected organ. Tissue placed in formalin was then paraffin-embedded and sliced into 5 micron sections. These were then mounted and subjected to hematoxylin and eosin (H&E) staining and subsequent imaging.

### PBMC immunological transcription assay

Blood collected from animals at 9 day-post inoculation (dpi) was used for the collection of peripheral blood mononuclear cells (PBMCs). Blood was diluted 1:4 with PBS lacking Ca^2+^ and Mg^2+^. The blood was then layered onto a Ficoll-paque Premium 1.084 cushion. The samples were then centrifuged at 340 x g for 30 min. The PBMC/lymphocyte layer was collected and washed 3 times with PBS lacking Ca and Mg at 300 x g for 5 min each. The resulting cell pellets were collected in Trizol-LS and used for an immunological transcription assay. RNA was extracted through use of the Roche cellular RNA large volume kit in the MagNA Pure 96 instrument. cDNA was produced using the BioRad iScript system according to the manufacturer’s instructions. The immunological PCR array was performed as previously described [20–22]. Ct values were analyzed using delta-delta Ct.

### PRNT

Plaque reduction neutralization tests (PRNTs) were performed on serum collected prior to euthanasia. 80 PFU of rRom was incubated for 1 hour at 37°C with 2-fold dilutions of the final serum. Following this initial incubation, VeroE6 cells were incubated with this virus/serum mixture for 1 hour at 37°C. Subsequently the monolayers were overlaid with MEM 2% FBS 1% P/S and 0.6% tragacanth gum. After 7 days the monolayers were fixed with 10% formalin and stained with crystal violet. Serum dilutions corresponding to a 50% plaque reduction were calculated.

### Statistical analysis

Statistical analysis was performed using GraphPad Prism 5 and 8 software. Samples with multiple replicates are shown as mean ±SD, with the exception of organ virus load which is shown as geometric mean ±geometric SD. Growth kinetics were analyzed via 2way ANOVA and the Bonferonni multiple comparisons test to determine significance compared to rRom. Analyte levels from clinical chemistry and hematology measurements and organ virus load were analyzed via 1way ANOVA and Dunnett’s multiple comparison test to determine significance compared to rRom. Survival curves were analyzed utilizing the Mantel-Cox log rank test.

## Results

### Effects of Can Z and mutation V64G on rRom growth kinetics *in vitro*

**Fig 1:**
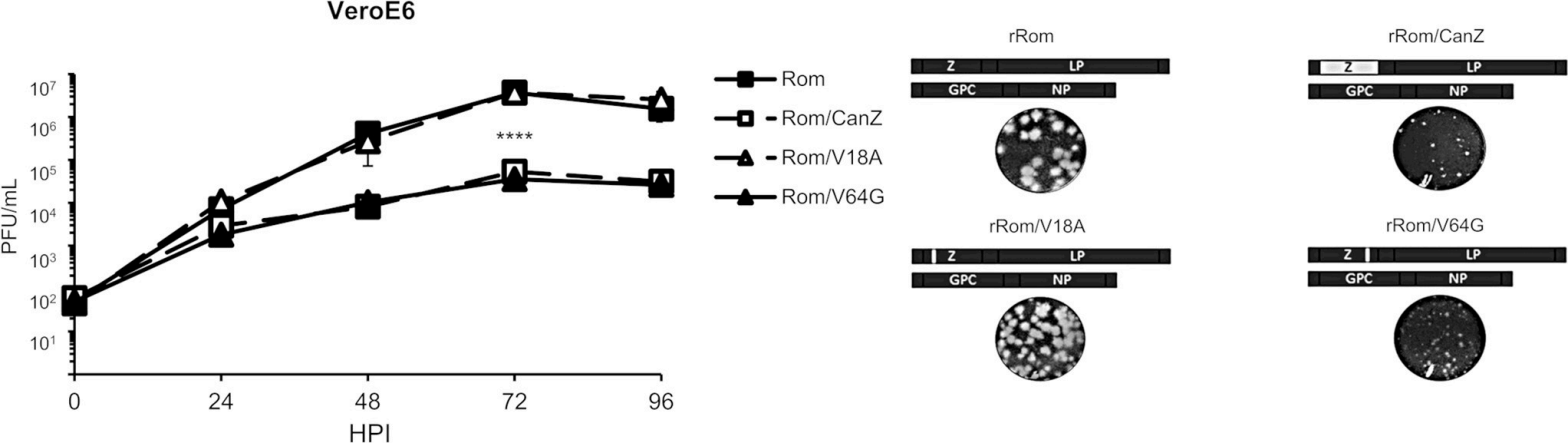
Analysis of rJUNV growth kinetics. VeroE6 cells were infected with each virus at MOI of 0.01. Supernatants were collected at 0, 24, 48, 72, and 96 hpi. Titers were determined by plaque assay on VeroE6 cells. Representative plaque morphology is shown for each virus, with large plaques shown for rRom and rRom/V18A and small plaques shown for rRomLP/CanZ and rRom/V64G. Samples were performed in triplicate. Error bars show the standard deviation. ** = p < 0.01, *** = p < 0.001 **** = p < 0.0001.

We have previously shown the growth kinetics of both rRom and rCan viruses, as well as inter-segment Rom/Can chimeric viruses, in both A549 and Vero cells. All viruses produced similar amounts of infectious particles at each time point [5]. However, characterization of rRom with a Can Z (rRom/CanZ) exhibited a significant reduction in growth kinetics during a multi-step growth assay in Vero E6 cells (Fig 1). We generated rRom containing each individual amino acid difference between Rom and Can Z proteins (rRom/V18A and rRom/V64G) and examined their growth kinetics in Vero cells following infection at MOI of 0.01 (Fig 1). As with rRom/CanZ, we found that rRom/V64G but not rRom/V18A, exhibited reduced growth kinetics when compared to rRom. In addition, both rRom/CanZ and rRom/V64G exhibited a similar pin-point plaque phenotype, whereas plaques produced by rRom/V18A were similar to those produced by rRom (Fig 1).

### Effect of Can Z and mutation V64G on rRom attenuation in Hartley guinea pigs (HGP)

**Fig 2:**
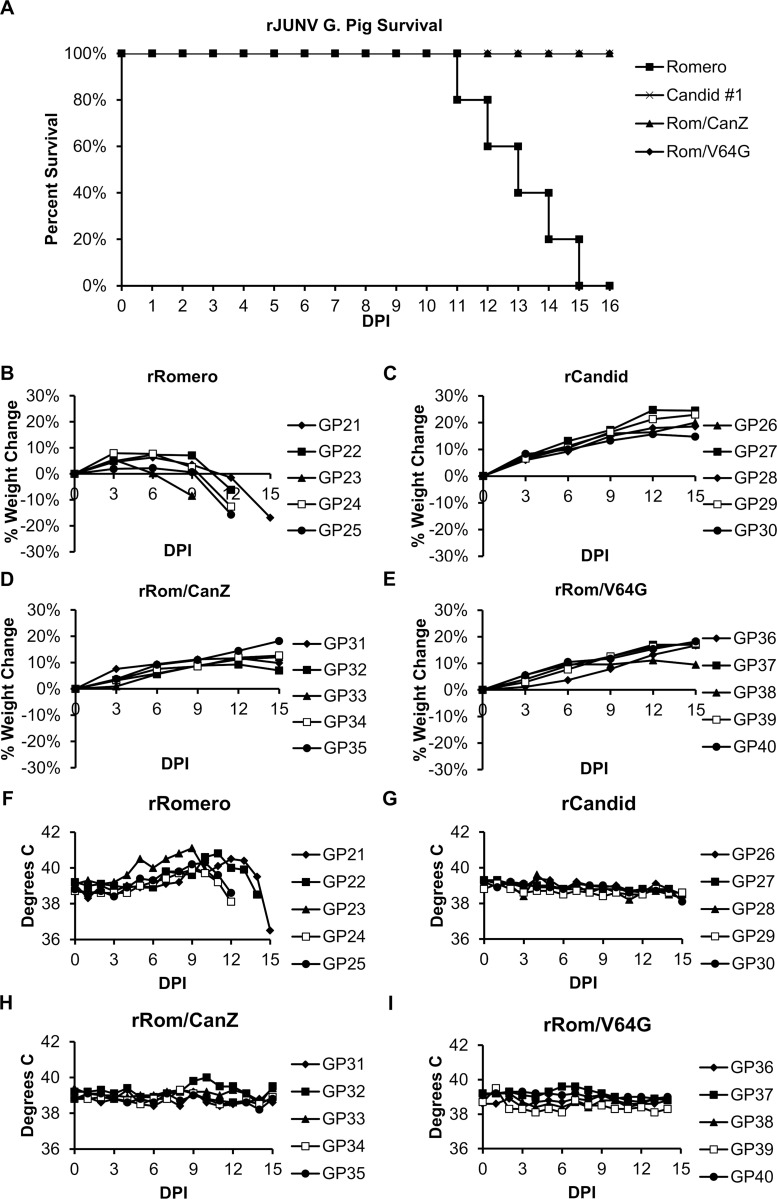
Guinea Pig survival, weight change, and temperature. Four groups of five animals each were infected with either rRom, rCan, rRom/CanZ, or rRom/V64G. Animals were monitored for weight, temperature, and clinical signs of disease. Panel A shows the survival curves of each group by day post infection. Since rCan, rRom/CanZ, and rRom/V64G infected animals all resulted in 100% survival, the curves are separated for ease of viewing. Panels B-E demonstrate the percent weight change from start of study. Panels F-I demonstrate the daily body temperature of each animal.

We next examined whether rRom/CanZ and rRom/V64G were, compared to rRom, attenuated *in vivo* using the well-established Hartley guinea pig (HGP) model of JUNV infection. We infected five HGP with 10^3^ PFU of each rRom, rCan, rRom/CanZ, or rRom/V64G and monitored them for the appearance of clinical symptoms including weight loss and fever for 15 days (Fig 2). All HGP inoculated with rCan, rRom/CanZ, and rRom/V64G survived and did not develop noticeable clinical symptoms with exception of one HGP inoculated with rRom/CanZ that exhibited transient fever. In contrast, all HGP inoculated with rRom exhibited weight loss and fever associated with progression of disease and succumbed to infection by 15 dpi. Animals in the surviving groups were maintained several days past the euthanasia of the final rRom infected animal to ensure that no disease symptoms developed at a delayed kinetic. While this does result in a non-matched final timepoint, we believe that absence of symptoms during the last 3 days in surviving animals likely indicates that the additional time did not change the final results. At the necropsy of rRom-inoculated guinea pigs, nasal bleeding, hemorrhage from the gastro-intestinal tract, small spleen, and petechial hemorrhage in liver were observed.

### Hematology measurements in HGP infected with rRom/CanZ or rRom/V64G

**Fig 3:**
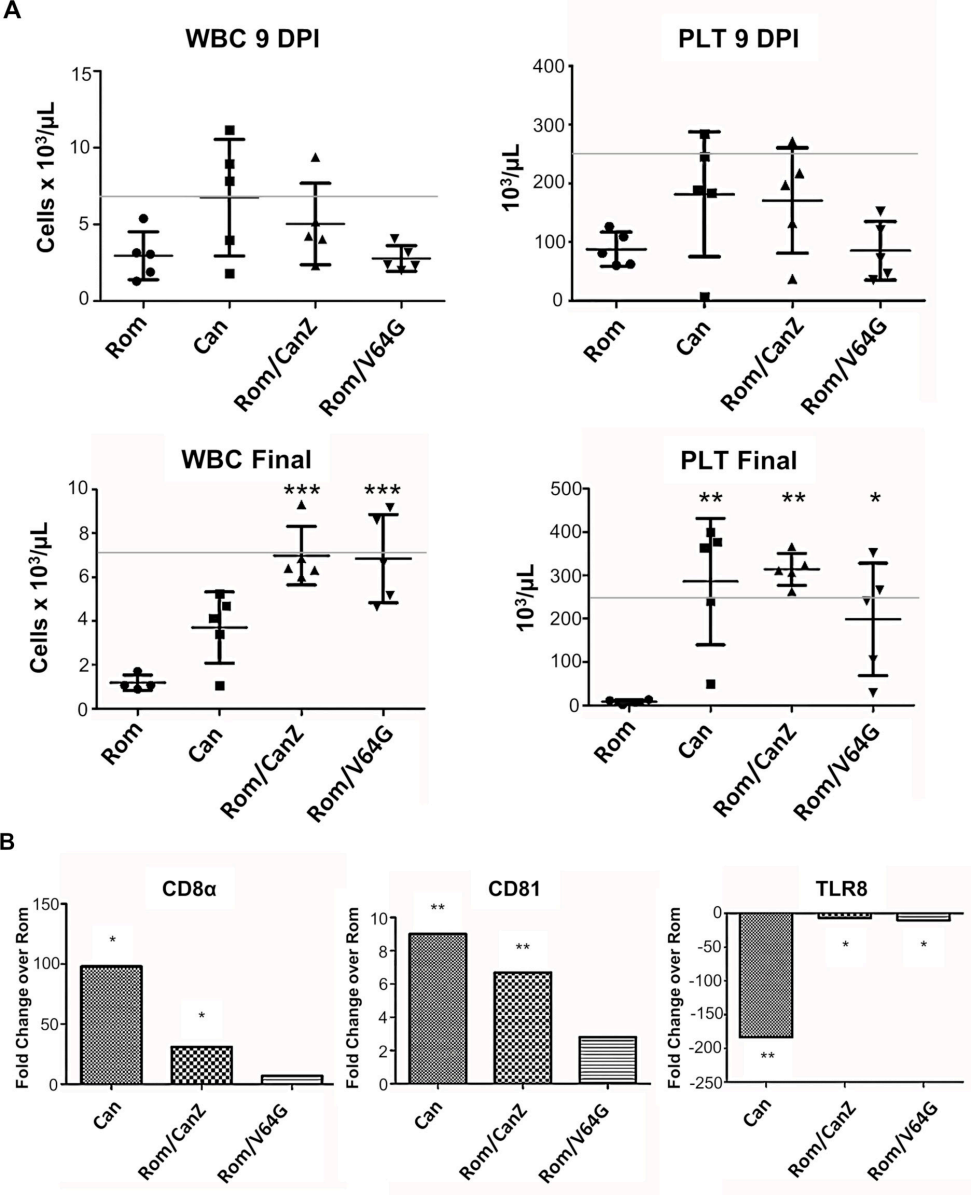
Guinea Pig hematology and immunological PCR array. Panel A shows blood was collected from all animals at 9 dpi and at the end of the study. Final blood collection from rRom infected animals came on the day of euthanasia (11–15 dpi) while final collection from all other groups occurred 17 dpi. Error bars represent the standard deviation. The gray line indicates the lower bound of normal levels for healthy Hartley guinea pigs. Panel B shows PBMCs collected 9 dpi were used for RNA isolation and qRT-PCR analysis of CD8α, CD81, and TLR8 mRNAs. rCan, rRom/CanZ, and rRom/V64G samples were compared to rRom samples to determine fold changes. * = p<0.05, ** = p < 0.01, *** = p < 0.001.

The animals in the study were subjected to blood collections at 9 dpi and prior to euthanasia. Whole blood was used to collect hematology data, and serum samples were used to determine blood clinical chemistry data. rRom and rRom/V64G infected animals demonstrated white blood cell (WBC) and platelet (PLT) counts below normal levels at 9 dpi, although these were not significantly different from the other groups (Fig 3A). By the final time point, however, both rRom/CanZ and rRom/V64G infected animals had returned to approximately normal counts for both parameters. Interestingly, rCan infected animals ended the study with WBC counts lower than normal, but above those of rRom animals. Consistent with the progression of hemorrhagic fever, rRom infected animals ended the study with nearly undetectable platelet levels.

### Comparison of immunological PCR array profiles of HGP infected with rRom, rCan, rRom/CanZ or rRom/V64G

Peripheral blood mononuclear cells (PBMCs) were isolated from whole blood samples of HGP collected at 9 dpi. The RNA isolated from PBMCs was analyzed using an established immunological PCR array. Values were normalized to housekeeping genes and expressed as fold changes over rRom infected samples. In total, 96 targets were analyzed. rCan and rRom/CanZ infected samples demonstrated significantly higher RNA levels of CD8α (CD8^+^ T cell marker) than rRom-infected samples. rRom/V64G infected samples did not exhibit a significant difference compared to rRom infected samples. However, both rRom/CanZ and rRom/V64G infected samples exhibited CD8α RNA levels lower than those found in rCan infected samples. Additionally, RNA levels of CD81 (antigen presenting cell marker) were significantly higher in rCan and rRom/CanZ infected samples. Finally, all groups showed a decreased RNA levels of TLR8 (ssRNA receptor), with rCan-infected samples showing the greatest reduction (Fig 3B).

The PCR array also included a target for JUNV genomic RNA to determine the presence of JUNV in the sample. PBMC samples collected at 9 dpi exhibited detectable levels of JUNV RNA in 4 of 5 rRom infected HGP, whereas none of the samples from rCan, rRom/CanZ, and rRom/V64G infected animals contained detectable levels of JUNV RNA by 9 dpi. Full results from PCR array are shown in S1 Table.

### Clinical chemistry measurements in infected HGP

**Fig 4:**
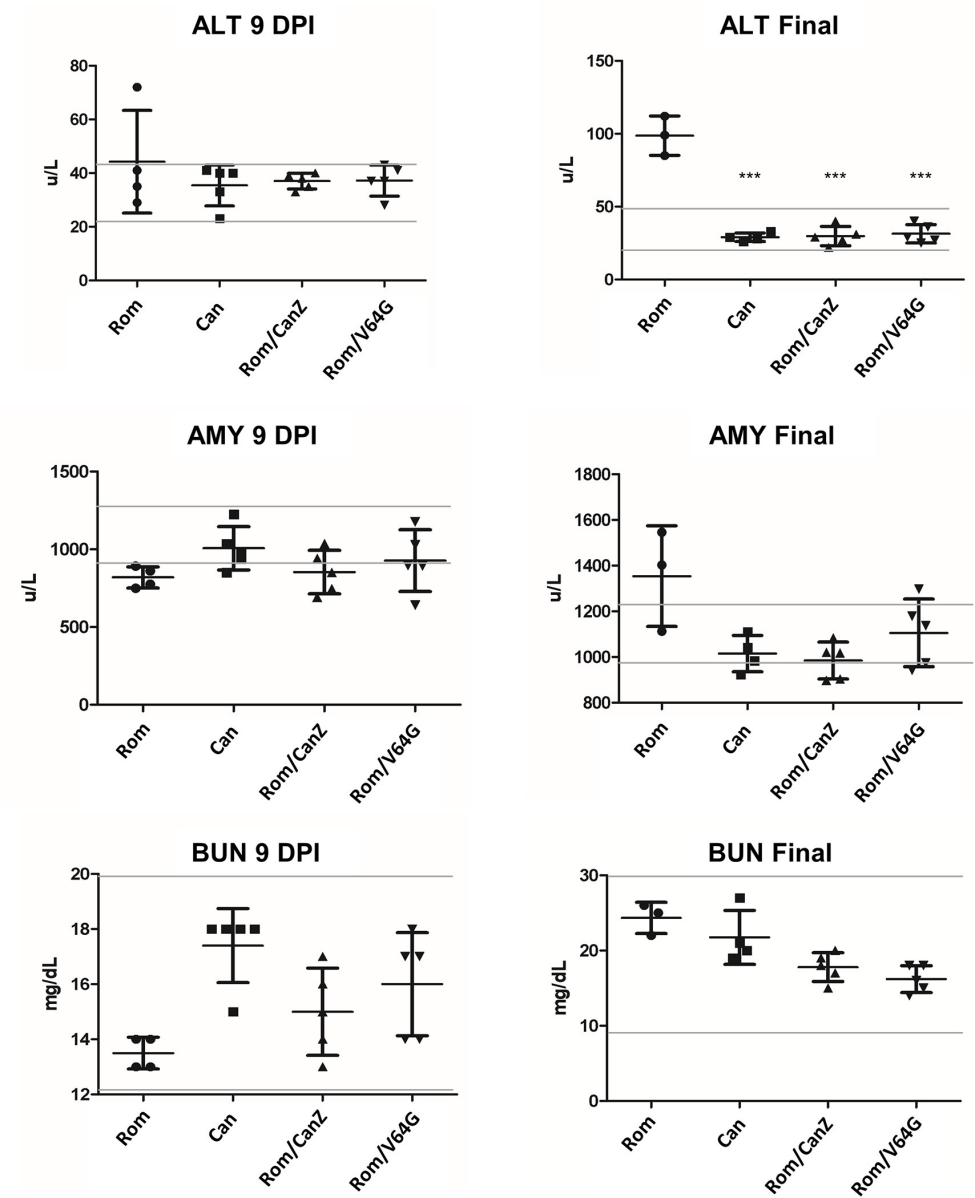
Guinea Pig clinical chemistry. Serum was collected from all animals at 9 dpi and at the end of the study. Final serum collection from rRom infected animals came on the day of euthanasia (11–15 dpi) while final collections from all other groups occurred 17 dpi. All groups were compared against rRom animals, and statistical significance is indicated by Asterix. The gray lines indicate the upper and lower bounds of normal levels for healthy Hartley guinea pigs. * = p < 0.05, ** = p < 0.01, *** = p < 0.001.

Analysis of blood chemistry also yielded valuable information about the pathogenesis of each of these viruses. We compared 3 separate analytes: alanine transferase (ALT), an indicator of hepatic inflammation and damage; amylase (AMY), an indicator of pancreatic inflammation and damage and blood urea nitrogen (BUN) as indicator of renal disfunction. At 9 dpi most HGP were within the normal analyte ranges, with the exception of rRom-infected animals that exhibited slightly reduced AMY levels (Fig 4). At the final blood collection, rRom-infected HGP exhibited significantly elevated ALT and AMY levels. While the BUN levels in rRom-infected HGP were significantly increased over rRom/CanZ and rRom/V64G infected HGP, they were still within the normal biological range.

### Viral load in tissues from HGP infected with rRom/CanZ or rRom/V64G

**Fig 5:**
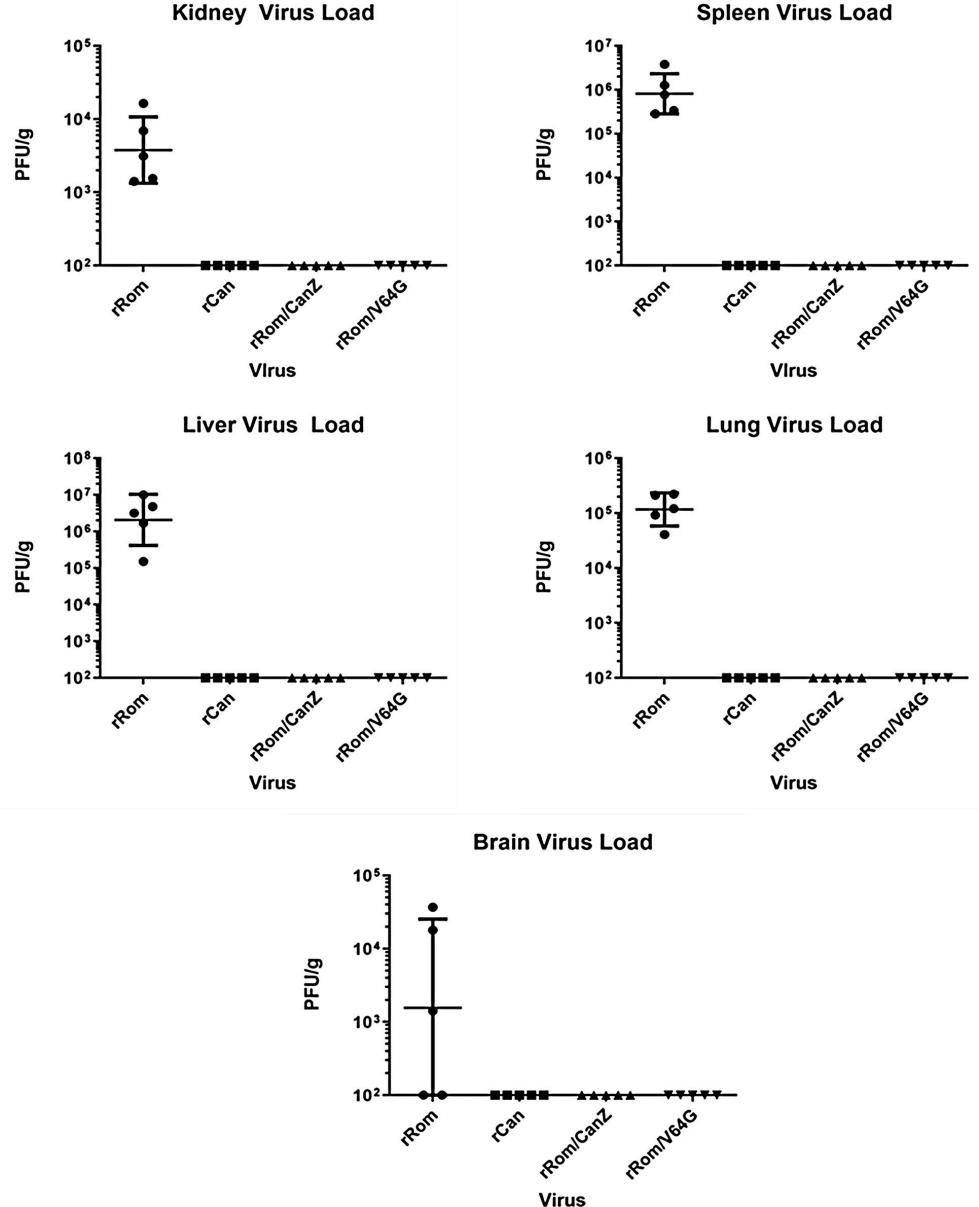
Guinea Pig organ viral loads. Following euthanasia or death, kidney, spleen, liver, lung, and brain samples were collected from all animals. Organs collected from rRom infected animals came on the day of euthanasia (11–15 dpi) while final collections from all other groups occurred 17 dpi. Samples are shown as geometric mean ±SD. All groups were compared against rRom animals and statistical significance is indicated by asterix. The limit of detection was 100 PFU/g and forms the lowest point on the Y axis. * = p < 0.05, *** = p < 0.001.

One of the hallmarks of the Can is its limited organ dissemination within infected hosts [23]. Accordingly, we examined whether rRom/CanZ and rRom/V64G infection exhibited also restricted organ dissemination by determining viral loads in kidney, spleen, liver, lung, and brain of infected HGP. Only rRom infected HGP had detectable viral load in all 5 organs (Fig 5).

### Plaque reduction neutralization (PRNT) antibody titers and organ histology in infected HGP

Serum from the final blood collections from infected HGP in PRNT assay with rRom. We could not collect enough serum to perform the assay for all animals. We tested serum samples from all 5 rRom, 2 rCan, 2 rRom/CanZ, and 3 rRom/V64G infected HGP. We did not detect neutralizing activity in any rRom-infected serum sample, while all other samples had neutralizing titers ranging from 1:5–1:24.

**Fig 6:**
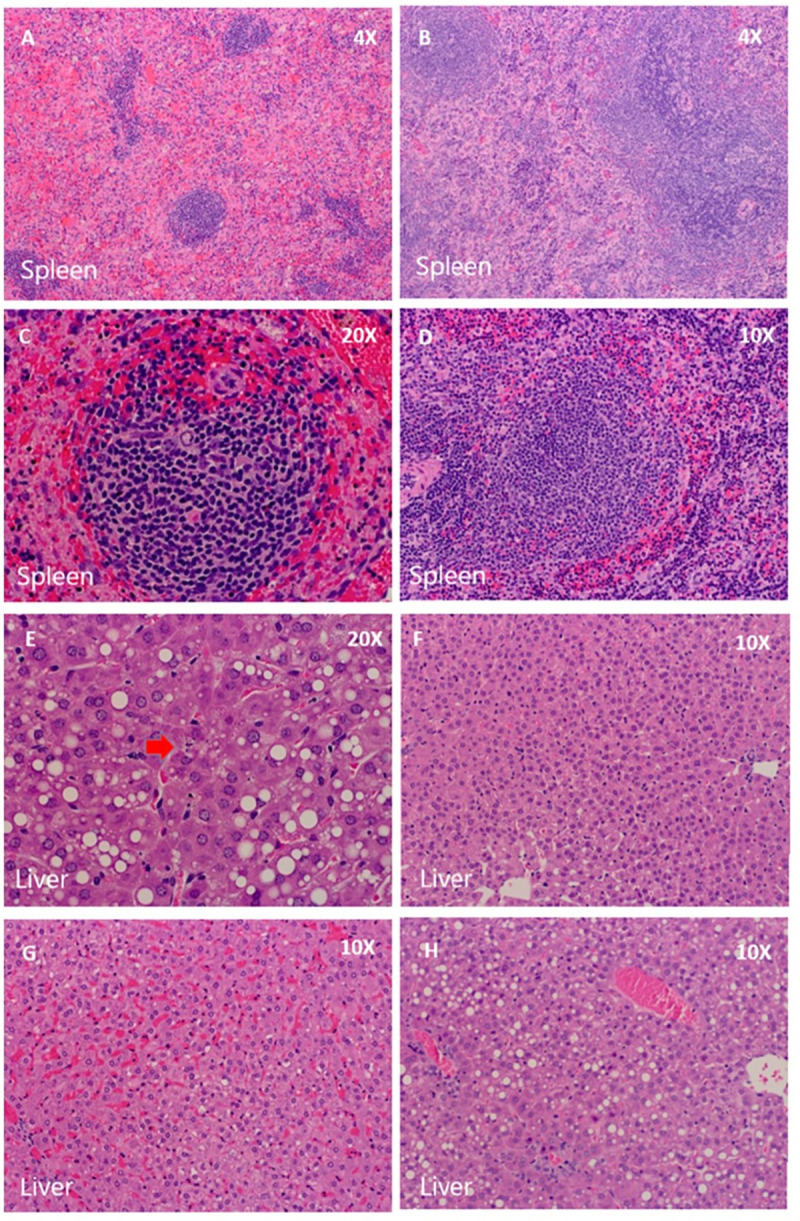
Organ histology. Spleen and Liver samples from rRom, rRom/CanZ, and rRom/V64G infected animals were fixed, embedded, mounted, and stained by H&E. Representative fields were imaged for each group. Spleens are shown for rRom (A and C), rRom/CanZ (B), and rRom/V64G (D). Livers are shown for rRom (E and G), rRom/CanZ (F), and rRom/V64G (H). Magnifications are shown in the upper right of each panel.

Histopathological assessment showed that rRom, but not rRom/CanZ or rRom/V64G, infected HGP exhibited significant spleen and liver damage. The rRom infected spleens demonstrated reduced white pulp and increased congestion in the red pulp. Neither rRom/CanZ nor rRom/V64G infected spleens exhibited any apparent pathology. The rRom infected livers exhibited fatty metamorphosis and apoptotic nuclear fragmentation as seen in the sinusoid space. A single rRom/V64G infected liver exhibited fatty metamorphosis, but neither rRom/CanZ nor rRom/V64G infection resulted in apoptotic nuclear fragmentation. Brain damage was not detected in any of the examined infected HGP (Fig 6).

## Discussion

The development of vaccines and therapeutics for viral hemorrhagic fevers is a pressing concern for global public health. The *Mammarenavirus* genus contains several important human pathogens including Lassa virus, JUNV, MACV, GTOV, and SABV, all of which can cause severe hemorrhagic fever disease in humans. Previous studies have shown that GPC is a main determinant of JUNV virulence as rRom /Can GPC exhibits the attenuated phenotype of Can [5,24,25]. Can GPC mediated attenuation of JUNV correlates with ablation of a conserved glycosylation motif in GPC that results in altered GPC trafficking and increased cellular stress [6]. However, there is a lack of knowledge about additional viral determinants of JUNV attenuation. In the present work we have shown that a single mutation (V64G) in the RING domain of the virus matrix Z protein was sufficient to reduce viral replication *in vitro* and attenuate the virus *in vivo* of rRom. At 9 dpi guinea pigs infected with rRom/V64G or rRom exhibited similar reductions in WBC, PLT counts and CD8α levels at 9 dpi, but WBC and PLT levels recovered in guinea pig infected with rRom/V64G. Guinea pigs infected with rCan or rRom/CanZ exhibited WBC and PLT counts and CD8α levels within the normal biological ranges. These findings are consistent with reports of decreased CD8^+^ T cell levels in AHF patients [26]. The rRom/CanZ exhibited a Can-like phenotype, indicating that the combination of both V18A and V64G mutations results in a stronger attenuating phenotype than the one associated with the single V64G mutation. Z interacts with LP [12–14,27], NP [28,29] and GPC [11], but whether V18A and V64G mutations influence these interactions remains to be determined. It is noteworthy that an inter-segment chimeric rCanL/RomS virus maintains similar growth kinetics to both rRom and rCan [5], suggesting that an incompatibility between Can Z and Rom LP might be the cause of the decreased infectious particle production for rRom/CanZ and rRom/V64G. It is also plausible that these Z mutations could affect the activity of the vRNP or assembly of infectious progeny, or both. The effect of the V64G mutation was readily apparent in cultured cells as decreased growth kinetics, whereas mutation V18A did not exhibit a noticeable phenotype in cultured cells. All surviving animals cleared the virus, which prevented us to assess potential differences between rRom/CanZ and rRom/V64G in their ability to disseminate *in vivo*. However, blood chemistry, hematology, and transcriptome results indicated that rRom/V64G infection resulted in clinical symptoms at early stages of infection, but no viral RNA was detected in PBMCs of rRom/V64G-infected guinea pigs. These results suggest that although rRom/V64G spreads more than rRom/CanZ early in infection and causes clinical manifestations similar to those associated with rRom infection, rRom/V64G is eventually controlled and cleared by the host immune response due to its impaired multiplication compared to rRom.

The closest parental strain to Can is the 13^th^ passage of strain XJ (XJ13), and XJ13 already has V18A and V64G Z mutations [30]. Whether these changes represent natural differences between XJ and Rom or whether they were mutations acquired during the first 13 passages of XJ strain is unknown, however the glycine at position 64 is not seen in any other sequenced JUNV strain (S1 Fig). While the possibility of reversion at this single amino acid position exists, we do not anticipate this as being likely since serial passage of the recombinant virus does not result in reversion (S2 Table).

JUNV is the only HF-causing mammarenavirus for which there is a live attenuated vaccine, Can, with demonstrated safety and efficacy in humans. Therefore, understanding the attenuating mechanisms of Can might facilitate future vaccine development for related hemorrhagic fever-causing NW mammarenaviruses. The RING domain present in the matrix Z protein is highly conserved among mammarenaviruses, raising the possibility that counterpart mutations to the V64G attenuating mutation we have documented here for JUNV might attenuate the closely related MACV, GTOV, and SABV. While it has been shown that mammarenaviruses are able to replicate in cell culture while lacking a Z [31], the introduction of Z incompatibilities as an attenuating strategy is warranted. The ability to introduce amino acid substitutions into the RING domain of the Z protein to impact viral growth, in combination with known attenuating mutations within the virus GPC, can significantly contribute to rationally designing live attenuated virus (LAV) candidates for highly pathogenic NW mammarenaviruses.

## Supporting information

S1 Table PBMC Immunological PCR array. PBMCs were collected 9 dpi and used for RNA isolation and qRT-PCR analysis. Results are shown as the average fold change over rRom infected samples. Empty cells indicate no amplification for the given target.
(PDF)

S2 Table Passage of rRom/V64G in VeroE6 cells. VeroE6 cells were infected with rRom/V64G at an MOI of 0.01 and allowed to incubate for up to 96 hours. The supernatant was collected and titrations were performed using plaque assay at various time points throughout infection. This process was repeated for a total of 7 passages, and the resulting virus was sequenced using Sanger sequencing. The peak titers for Passage 1 and Passage 7 are shown for rRom/V64G. Passage 1 peak titers for rRom and rRom/V18A are provided for comparison. Plus-minus values represent the standard deviation values among triplicate repeats.
(TIF)

S1 Fig Alignment of known arenavirus Z sequences. Z protein open reading frame sequences were taken from GenBank and aligned using MUSCLE. Results were viewed using JalView, and color coding was provided to each amino acid based on the properties of the R group. Only XJ13, XJ44, and Can Z have a Gly-like residue at JUNV position 64 (marked with red arrow).
(TIF)
