# Supplementary material for: A single mutation (V64G) within the RING Domain of Z attenuates Junin virus
Source: PLoS Negl Trop Dis. 2020 Sep 25;14(9):e0008555. doi: 10.1371/journal.pntd.0008555 (PMC7540883; doi:10.1371/journal.pntd.0008555)
Supplement: S1 Table — PBMCs were collected 9 dpi and used for RNA isolation and qRT-PCR analysis. Results are shown as the average fold change over rRom infected samples. Empty cells indicate no amplification for the given target. (PDF) [file pntd.0008555.s001.pdf]

Target	rCan vs rRom Fold Change	rRom/CanZ vs. rRom Fold Change	rRom/V64G vs. rRom Fold Change
TLR-2	1.2	5.8	1.3
TLR-3	-11.2	-4.1	1.9
TLR-4	-47.9	-13.5	-10.8
TLR-6	-1.5	1.7	3.5
TLR-7	10.6	3.0	13.4
TLR-8	-183.7	-6.8	-10.1
TLR-9	-3.7	-2.8	2.4
TLR-10	1.2	8.1	1.2
CD4	2.3	1.9	1.1
CD8alpha	97.8	30.8	6.8
CD62L	-2.4	-2.7	1.9
CD107a	-1.6	-1.2	-2.0
CD107b	1.1	-1.2	1.6
CD134	1.2	1.2	1.6
CD152	15.1	3.9	
CD223	8.0	7.3	9.1
CD126-1	-1.3	-1.4	-2.9
CD130	-1.0	-2.9	-1.5
CD19-2	5.0	10.2	1.6
CD22	30.4	19.3	11.5
CD79a	2.8	3.0	-1.3
CD79b	2.2	2.6	-1.6
CD20(2)	2.8	2.3	2.2
CD94	2.4		
CD23	2.9	-1.8	3.8
CD25	2.3	1.4	9.0
CD39	3.3	5.7	
CD69	3.7	10.6	1.2
CD72	1.0	2.5	-2.2
CD92	1.4	-1.4	-1.5
CD93(2)	3.1	1.2	2.8
CD115	3.5	4.8	-1.4
CD2	12.3	10.3	1.1

IFN gamma	3.5	2.0	5.6
IFNAR1	2.8	-6.2	1.2
IFNGR1(2)	-21.2	-4.3	-11.1
GM-CSF		1.7	
LTA	1.5	9.9	3.0
lysozyme	-4.5	-1.7	-2.1
KLRG1	17.7	27.5	
beta2 u-globin	1.5	1.5	-19.9
MHC-II	-1.2	4.0	1.3
CIITA	24.5	27.0	3.8
RANTES	35.8	22.9	2.4
MCP-1	-5.0	-9.9	10.9
Fc gamma1/2 receptor	-1.9	1.0	-1.2
NFkB1	-1.1	1.1	-1.4
IL-15(3)	1.5	-1.1	2.0
CXCL10	-758.1	-5.8	-16.1
CXCL11	5.1		
GNCP-1b	-70.9	-40.0	-45.7
IL-1b	15.7	1.5	5.8
TNFSF4	-1.0		
CCR6-1	-1.3	3.0	-3.0
CD36(2)	-1.1	3.2	1.6
CD180	7.2	27.7	7.5
CD28	1.3	-2.4	-2.4
CD40	11.3	3.5	1.9
CD44	-1.0	-2.0	-2.8
CD81	9.0	6.7	2.8
SOD1	1.5	1.1	1.0
IFNAR2	-1.4	-2.2	-5.1
CD96	10.8	1.1	8.7
IL-12p40	12.8	58.1	14.6
IL-4 receptor	-1.4	-1.7	-6.1
IL-16	6.3	1.1	8.5
IL-23 receptor		2.0	

IL-27	-11.5	-2.6	1.1
IL-27 receptor a	12.2	4.3	1.7
CD14	-2.5	-2.2	-4.6
IL-7 receptor	-2.3	-1.2	-1.4
IL-18	-1.0	-3.8	-5.0
IL-21	4.5		
CCR3 receptor	1.4	1.4	2.8
IL-8	-1.2	1.9	1.0
SOD2	-1.2	-1.1	-1.0
TGFbeta	-1.5	-1.4	-3.8
TNFalpha	1.6	-2.2	1.5
CXCL12	-3.2	-2.4	1.5
CCR4 receptor	2.1	1.7	
CXCR1	-4.6	-3.2	-4.5
CXCR3	14.3	1.7	6.5
CXCR2 IL-8 receptor beta	-1.8	-2.1	-4.5
IL-5 receptor beta chain	-17.0	-4.6	-24.6
