# Supplementary figures and images for: A single mutation (V64G) within the RING Domain of Z attenuates Junin virus
Source: PLoS Negl Trop Dis. 2020 Sep 25;14(9):e0008555. doi: 10.1371/journal.pntd.0008555 (PMC7540883; doi:10.1371/journal.pntd.0008555)

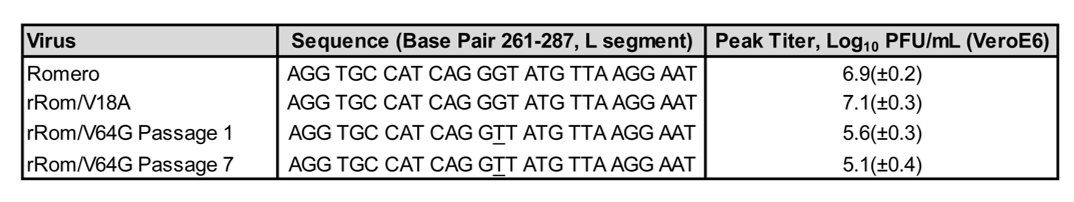

Supplement: S2 Table — VeroE6 cells were infected with rRom/V64G at an MOI of 0.01 and allowed to incubate for up to 96 hours. The supernatant was collected and titrations were performed using plaque assay at various time points throughout infection. This process was repeated for a total of 7 passages, and the resulting virus was sequenced using Sanger sequencing. The peak titers for Passage 1 and Passage 7 are shown for rRom/V64G. Passage 1 peak titers for rRom and rRom/V18A are provided for comparison. Plus-minus values represent the standard deviation values among triplicate repeats. (TIF) [file pntd.0008555.s002.tif]

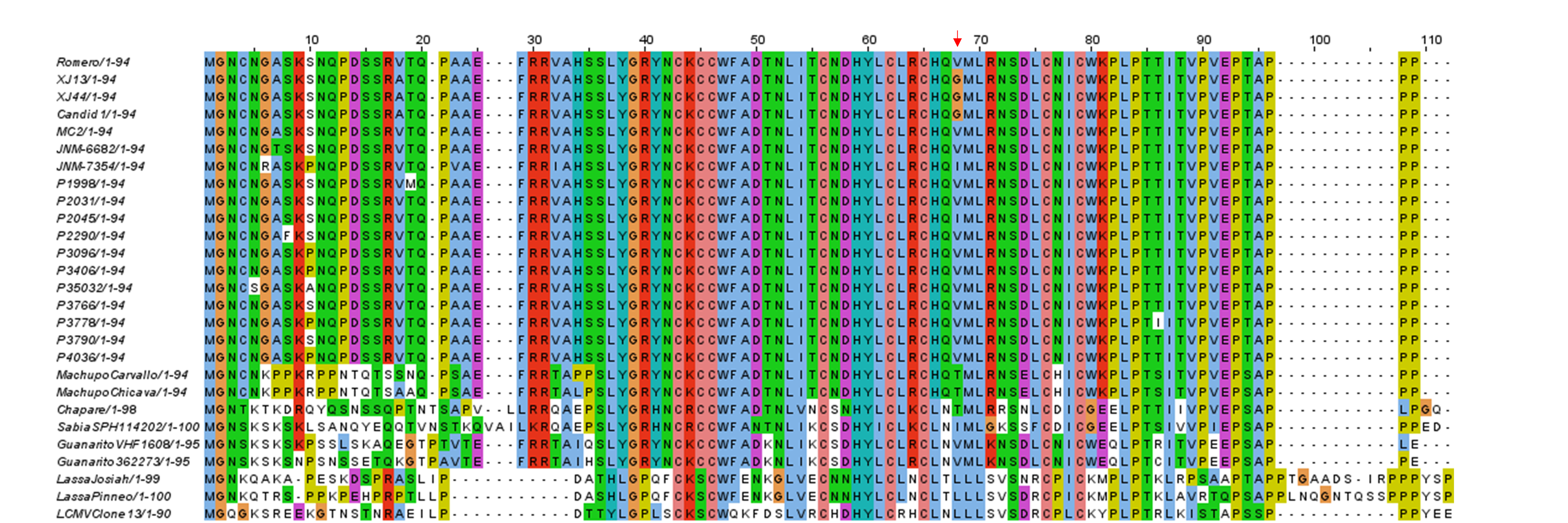

Supplement: S1 Fig — Z protein open reading frame sequences were taken from GenBank and aligned using MUSCLE. Results were viewed using JalView, and color coding was provided to each amino acid based on the properties of the R group. Only XJ13, XJ44, and Can Z have a Gly-like residue at JUNV position 64 (marked with red arrow). (TIF) [file pntd.0008555.s003.tif]
